# CT-based 3D bone shape modes are associated with clinically meaningful functional improvement after total knee arthroplasty

**DOI:** 10.1016/j.ocarto.2026.100839

**Published:** 2026-06-06

**Authors:** Wei Wang, Lawrence Chun Man Lau, John A. Lynch, Tianshu Jiang, Zhiqiang Wang, Lok Chun Chan, Henry Fu, Kwong Yuen Chiu, Thomas M. Link, David J. Hunter, Ping Keung Chan, Chunyi Wen

**Affiliations:** a Department of Biomedical Engineering, Faculty of Engineering, The Hong Kong Polytechnic University, Hung Hom, Hong Kong, China; b Department of Orthopaedics and Traumatology, Queen Mary Hospital, The University of Hong Kong, Pok Fu Lam, Hong Kong, China; c Department of Epidemiology and Biostatistics, University of California, San Francisco, CA, USA; d Department of Radiology and Biomedical Imaging, University of California, San Francisco, CA, USA; e Department of Rheumatology, Royal North Shore Hospital and Sydney Musculoskeletal Health, Faculty of Medicine and Health Science, Kolling Institute, University of Sydney, Sydney, Australia; f Research Institute of Smart Ageing, The Hong Kong Polytechnic University, Hung Hom, Hong Kong, China

**Keywords:** Total knee arthroplasty, Knee osteoarthritis, Minimal clinically important difference, Knee society function score, Statistical shape modeling

## Abstract

**Objective:**

To evaluate whether preoperative CT-derived three-dimensional bone shape modes are associated with failure to achieve the Knee Society Score Function subscore (KSS-Function) minimal clinically important difference (MCID) after total knee arthroplasty (TKA).

**Design:**

This retrospective study included patients with knee osteoarthritis who underwent primary robotic-assisted knee arthroplasty between January 2019 and January 2024. Bone shape modes were extracted from preoperative CT-based segmentations of the distal femur, proximal tibia, and patella using statistical shape modeling. Clinical outcome analysis was restricted to TKA knees with complete 1-year KSS-Function follow-up. MCID non-achievement was defined as failure to achieve a 10-point improvement in KSS-Function. Associations between shape modes and MCID non-achievement were assessed using logistic regression, with adjustment for clinical covariates. Internal discrimination was evaluated using repeated patient-level grouped cross-validation.

**Results:**

Among 151 TKA knees, 15.9% did not achieve the KSS-Function MCID. Female sex and higher BMI were associated with greater odds of MCID non-achievement. After adjustment for sex and BMI, five shape modes were associated with MCID non-achievement. These modes reflected less pronounced patellofemoral bony remodeling, patellar median ridge curvature, and localized contour variation near the central tibial plateau. The clinical-only, shape-only, and combined models achieved AUCs of 0.680, 0.696, and 0.754, respectively. The combined model had a 0.074 higher AUC than the clinical-only model, but paired bootstrap analysis was not significant (*p* = 0.130).

**Conclusions:**

CT-derived bone shape modes were associated with MCID non-achievement after TKA, suggesting bone morphology may offer insights, but their incremental value requires confirmation in larger, adequately powered cohorts.

## 1 Introduction

Total knee arthroplasty (TKA) is widely regarded as the definitive treatment for advanced knee osteoarthritis (OA), and its utilization continues to rise, with annual procedures in the United States projected to approach 3 million by 2026 as the population ages [1,2]. However, despite high rates of technical success, postoperative recovery remains heterogeneous, with 12.7%–21.9% of patients reported to be dissatisfied with their outcomes [[3], [4], [5], [6], [7], [8]]. Patient dissatisfaction after TKA is multifactorial, with pain relief, functional improvement, expectations, and psychological factors all contributing to patient-perceived outcomes. Among these dimensions, postoperative function is an important and clinically measurable domain of recovery [9,10]. Therefore, better understanding preoperative factors associated with postoperative functional recovery may help explain outcome variability after TKA.

The Knee Society Function subscore (KSS-Function) is a validated patient-reported outcome measure widely used to assess functional status after knee arthroplasty [11,12]. For KSS-Function, the minimal clinically important difference (MCID) has been established using patient satisfaction as an anchor to define clinically meaningful postoperative functional improvement [13,14]. Failure to achieve this threshold therefore represents a clinically relevant endpoint reflecting insufficient functional gain from the patient’s preoperative status.

Preoperative advanced imaging, such as CT and MRI, is increasingly used for disease assessment and surgical planning in knee OA. However, compared with the well-recognized prognostic value of clinical factors, the prognostic information embedded in these imaging data remains less well explored. Although prior studies have reported that radiographic severity is associated with patient-reported outcomes [8,15,16], a recent large-scale study found that radiograph-based machine-learning models showed limited performance compared with clinical data-based models [17], suggesting limited incremental value beyond clinical data. Compared with conventional radiographs, cross-sectional imaging such as CT provides high spatial resolution and detailed three-dimensional bony morphology [18,19], offering an opportunity to extract complementary anatomical information from imaging data already acquired for surgical planning.

In this context, statistical shape modeling (SSM) provides a quantitative framework for extracting this underused three-dimensional morphological information by representing complex bone geometry as principal modes of anatomical variation [20]. In knee OA research, SSM-derived shape features have been successfully applied to predict radiographic onset of OA [21,22], disease progression, pain, and functional limitations [23], as well as the risk of TKA [24]. Unlike categorical Kellgren–Lawrence grades, bone shape representations offer more granular and quantitative characterization of morphological variation.

Building on these advances, the purpose of this study was to evaluate whether preoperative CT-derived 3D bone shape modes provide anatomical information associated with 1-year KSS-Function MCID non-achievement after TKA. We further explored the internal cross-validated performance of clinical, shape-only, and combined models for identifying MCID non-achievement. We hypothesized that specific femoral, tibial, and patellar shape modes would be associated with failure to achieve clinically meaningful functional improvement after TKA.

## 2 Method

### 2.1 Study participants

This retrospective longitudinal study was approved by the institutional review board with a waiver of informed consent (approval number UW 22-090). Consecutive patients who underwent primary robotic-assisted total or unicompartmental knee arthroplasty (TKA or UKA) for knee osteoarthritis were included from January 2019 to January 2024 for the CT-based SSM construction. The clinical outcome analysis was restricted to TKA knees with available 1-year KSS-Function data. All TKAs were performed with functional alignment using a single implant (Triathlon CR, MAKO, Stryker) and patellar electrocauterization without resurfacing.

**Fig. 1:**
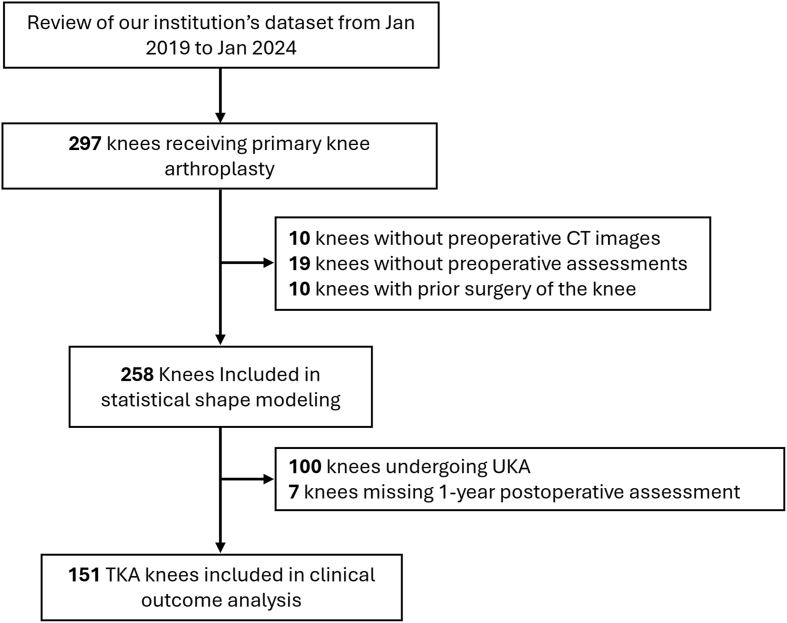
Patient inclusion flowchart.

Exclusion criteria included absence of preoperative CT imaging, missing KSS assessment, and prior knee surgery. Baseline demographic data, preoperative CT scans and KSS scores were collected, with follow-up KSS also obtained 1-year postoperatively (Fig. 1).

### 2.2 CT image processing and statistical shape modeling

**Fig. 2:**
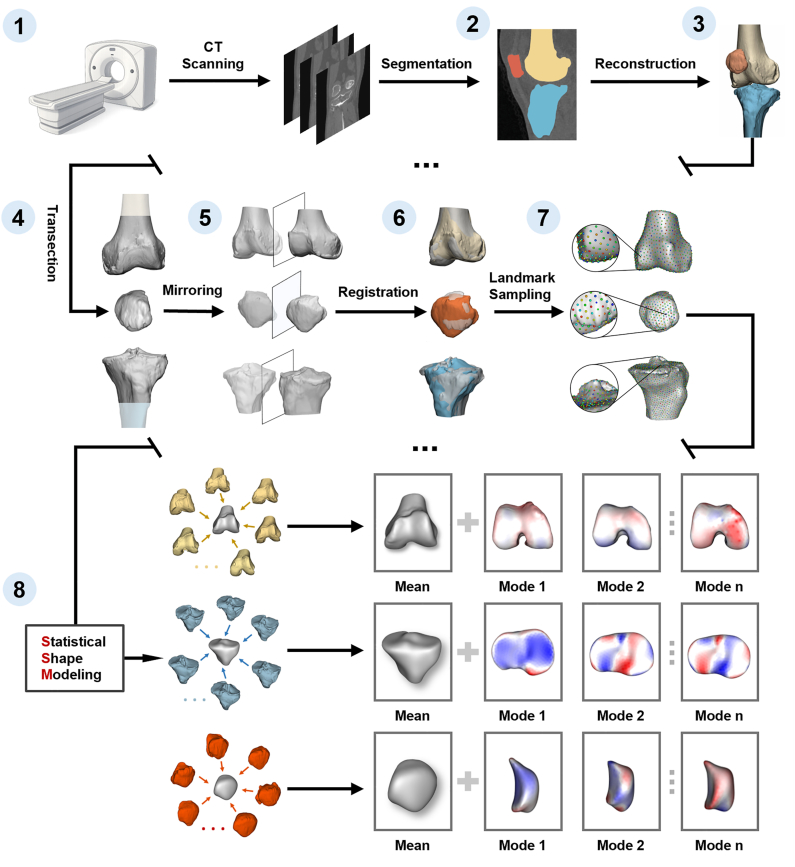
CT-based SSM workflow. (1) Preoperative CT scans were acquired. (2) The femur, tibia, and patella were segmented from CT images. (3) Three-dimensional surface models of the knee bones were reconstructed. (4) Bone regions of interest were defined by anatomical transection (see details in Fig. S1). (5) Right-sided knees were mirrored to the left to standardize anatomical orientation across all cases. (6) Bone surfaces were aligned to a randomly selected template surface using Procrustes registration followed by iterative closest-point registration. (7) Particle-based sampling was performed to establish point correspondence across subjects. (8) Statistical shape models were generated separately for the femur, tibia, and patella. Principal component analysis was then applied to derive the mean shape and dominant modes of shape variation for each bone. These SSM-derived shape mode scores were subsequently used as CT-derived anatomical features for outcome analysis.

CT images were acquired using a GE Revolution CT scanner (slice thickness: 0.625 mm; pixel spacing: 0.468 × 0.468 mm; matrix size: 512 × 512). As shown in Fig. 2, The distal femur, proximal tibia, and patella were semi-automatically segmented using 3D Slicer (version 4.10.2) and reconstructed as triangulated surface models. Regions of interest for the femur and tibia were defined relative to anatomical landmarks (Fig. S1). Right-sided knees were mirrored to the left to standardize anatomical orientation across all cases, after which meshes uniformly smoothed and remeshed.

SSMs of the femur, tibia, and patella were generated separately using ShapeWorks (V6.5.0) based on the entire arthroplasty dataset, including both TKA and UKA knees, to capture population-level variation in three-dimensional bone morphology among patients with advanced knee osteoarthritis. Surfaces were aligned using Procrustes registration [25] and the iterative closest-point algorithm, followed by particle-based correspondence optimization [26]. Principal component analysis was applied to the resulting point distribution models to extract dominant modes of shape variation. Shape modes explaining 90% of cumulative variance were retained as a predefined dimensionality-reduction criterion to preserve the dominant anatomical variation while limiting lower-variance modes, consistent with prior SSM practice [22]. Individual bone shapes were projected onto these modes to obtain knee-specific feature scores. For subsequent outcome analysis, only feature scores from TKA knees were used.

### 2.3 Clinical outcomes

For each patient, the KSS-Function subscale (0–100) were recorded preoperatively and at 1 year postoperatively. The primary outcome was achievement of clinically meaningful functional improvement, defined by the 1-year change in KSS-Function. Following published thresholds [14], patients who achieved a <10-point improvement were classified as MCID non-achievement, whereas those with a ≥10-point improvement were classified as MCID achievement.

### 2.4 Statistical analysis

Descriptive statistics were produced for the entire dataset used for SSM construction and for the TKA cohort used for clinical outcome analysis. Continuous variables were summarized as mean ± standard deviation or median with interquartile range, as appropriate, and categorical variables were summarized as counts and percentages. The association between baseline clinical variables, including age, sex, BMI, preoperative KSS-Knee, and preoperative KSS-Function, and 1-year MCID non-achievement was examined using univariable logistic regression.

The association between SSM-derived bone shape modes and MCID non-achievement were also assessed. All femoral, tibial, and patellar shape modes retained from the SSM analysis were first evaluated using univariable logistic regression, followed by multivariable logistic regression adjusted for clinical covariates associated with MCID non-achievement in the baseline clinical-variable analysis. Shape mode scores were standardized before analysis, and odds ratios with 95% confidence intervals were reported per one-standard-deviation increase in each shape mode score. To account for potential non-independence from patients contributing bilateral knees, regression analyses were performed with patient-level cluster-robust standard errors.

To evaluate the internal discriminative performance of CT-derived shape features for identifying MCID non-achievement, three logistic-regression-based models were constructed: a clinical-only model, a shape-only model, and a combined model. The clinical-only model included baseline clinical variables associated with MCID non-achievement. Given the limited number of MCID non-achievement events, the shape-only model was restricted to a parsimonious set of shape modes to reduce model complexity. Shape modes were eligible for inclusion in this parsimonious model if they remained associated with MCID non-achievement in both univariable and clinically adjusted logistic regression analyses with consistent effect directions. The combined model incorporated the selected clinical predictors and selected shape modes. The selected predictors were fixed before cross-validation, and no additional feature selection was performed during model evaluation.

Model discrimination was quantified using the area under the receiver operating characteristic curve (AUC). Internal model performance was assessed using repeated five-fold stratified grouped cross-validation (CV), repeated 100 times with different random seeds. Patient ID was used as the grouping variable to ensure that knees from the same patient were assigned to the same fold, thereby avoiding patient-level information leakage from bilateral knees. Within each training fold, predictors were standardized, and an L2-regularized logistic regression model with balanced class weights was fitted. The trained model was then applied to the corresponding held-out fold to generate out-of-fold predicted probabilities for MCID non-achievement.

For each repetition, AUC was calculated using the out-of-fold predicted probabilities. Repeated-CV AUCs were summarized as mean ± standard deviation, and the empirical 2.5th–97.5th percentile interval was calculated from the distribution of AUCs across the 100 repetitions. For ROC curve visualization, predicted probabilities were averaged across the 100 repetitions for each knee, and AUC was calculated using these mean cross-validated predicted probabilities.

The difference in AUC between the clinical-only model and the combined model was evaluated using paired patient-level bootstrap resampling based on the mean cross-validated predicted probabilities, with patients used as the resampling unit to preserve the correlation structure of bilateral knees. In each of 5,000 bootstrap iterations, AUCs were recalculated for both models, and the ΔAUC was recorded. A two-sided bootstrap P value was calculated based on the proportion of bootstrap ΔAUC values on the opposite side of zero. All analyses were conducted using Python 3.9.7 and R, with statistical significance defined as a two-sided P value < 0.05.

## 3 Results

### 3.1 Participant characteristics

A total of 258 knees were included for SSM-based extraction of shape features (see clinical characteristics in Table S1). Clinical outcome analysis included 151 knees from 109 patients with complete 1-year follow-up. Among them, 127 knees achieved MCID, and 24 knees did not achieve MCID, corresponding to an MCID non-achievement rate of 15.9%. Baseline clinical characteristics of the TKA outcome cohort, stratified by MCID achievement status, are presented in Table 1.

**Table 1:** Baseline clinical characteristics of the TKA outcome cohort stratified by MCID achievement status.

	Overall TKA cohort	MCID achievement	MCID non-achievement
Knees, n	151	127	24
Patients, n	109	89	22
Age at surgery, years	66.5 ± 5.9	66.3 ± 5.8	67.9 ± 6.3
Female sex, n (%)	96 (63.6%)	75 (59.1%)	21 (87.5%)
BMI, kg/m^2^	28.3 ± 4.7	27.9 ± 4.4	30.5 ± 5.7
Preop KSS-knee	53.5 ± 16.2	53.1 ± 16.5	55.9 ± 14.5
Preop KSS-function	48.9 ± 13.1	48.8 ± 13.1	49.2 ± 13.8

**Note:** Continuous variables are presented as mean ± standard deviation, and categorical variables are presented as frequency (%).

**Abbreviations:** MCID, minimal clinically important differences; TKA, total knee arthroplasty; BMI, body mass index; KSS-Knee, Knee Society Knee Score; KSS-Function, Knee Society Function Score.

### 3.2 CT-based shape feature extraction

**Fig. 3:**
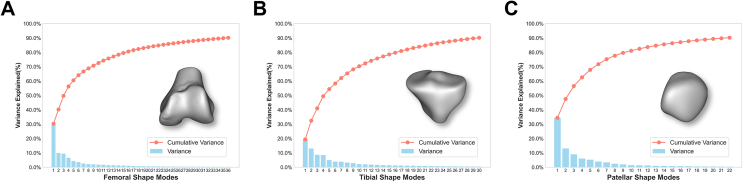
Retained shape modes using cut-off method. A. Thirty-six shape modes for the femur, B. thirty modes for the tibia, and C. twenty-two modes for the patella were initially retained using cut-off method, each capturing 90% of the respective shape variances in SSM.

In the statistical shape analysis, the first 36 femoral, 30 tibial, and 22 patellar principal components were retained, each capturing 90% of cumulative shape variance for the corresponding bone (Fig. 3). These retained components were used as SSM-derived shape feature scores in the subsequent TKA outcome analysis.

### 3.3 Clinical variables associated with MCID non-achievement

Univariable logistic regression results for baseline clinical variables are presented in Table 2. Male sex was associated with lower odds of MCID non-achievement compared with female sex (OR, 0.206; 95% CI, 0.056–0.763; *p* = 0.018), whereas higher BMI was associated with greater odds of MCID non-achievement (OR, 1.665 per 1-SD increase; 95% CI, 1.060–2.615; *p* = 0.027). No significant associations were observed for age, preoperative KSS-Knee, or preoperative KSS-Function.

**Table 2:** Univariable logistic regression analysis of clinical variables associated with 1-year KSS-Function MCID non-achievement after TKA.

Variables	Odds ratio (95% CI)	P value

Sex	0.206 (0.056–0.763)	**0.018**
BMI	1.665 (1.060–2.615)	**0.027**
Age	1.321 (0.788–2.216)	0.291
Preoperative KSS-Knee	1.204 (0.755–1.920)	0.435
Preoperative KSS-Function	1.027 (0.673–1.569)	0.901

**Note:** The outcome was 1-year KSS-Function MCID non-achievement after TKA, coded as 1 for non-achievement and 0 for achievement. Sex was coded as female = 0 and male = 1. Continuous variables were standardized; odds ratios are reported per 1-SD increase. Patient-level cluster-robust standard errors were used to account for bilateral knees.

**Abbreviation:** MCID, minimal clinically important differences; TKA, total knee arthroplasty; BMI, body mass index; KSS-Knee, Knee Society Knee Score; KSS-Function, Knee Society Function Score.

### 3.4 Bone shape modes associated with MCID non-achievement

All femoral, tibial, and patellar shape modes retained from the SSM analysis were evaluated for their associations with 1-year KSS-Function MCID non-achievement. Nine candidate shape modes associated with MCID non-achievement in either unadjusted or sex- and BMI-adjusted analyses are summarized in Table 3. After adjusted for sex and BMI, femoral mode 14 and tibial mode 19 were associated with higher odds of MCID non-achievement, with adjusted ORs of 1.626 (95% CI, 1.012–2.614; *p* = 0.045) and 1.714 (95% CI, 1.046–2.811; *p* = 0.033), respectively. In contrast, patellar mode 3, patellar mode 16, and tibial mode 26 were associated with lower odds of MCID non-achievement, with adjusted ORs of 0.571 (95% CI, 0.326–1.000; *p* = 0.050), 0.469 (95% CI, 0.280–0.786; *p* = 0.004), and 0.613 (95% CI, 0.392–0.960; *p* = 0.032), respectively.

**Table 3:** Unadjusted and sex- and BMI-adjusted associations between bone shape modes and 1-year KSS-Function MCID non-achievement after TKA.

Shape mode	Variance explained (%)	Unadjusted OR (95% CI)	*p* value	Adjusted OR (95% CI)	P value

Femoral mode 14	1.3%	1.290 (0.860–1.935)	0.218	1.626 (1.012–2.614)	**0.045**
Femoral mode 18	0.7%	1.439 (1.009–2.050)	**0.044**	1.249 (0.833–1.874)	0.282
Femoral mode 32	0.3%	0.582 (0.398–0.851)	**0.005**	0.682 (0.461–1.008)	0.055
Patellar mode 3	9.0%	0.615 (0.378–1.001)	0.051	0.571 (0.326–1.000)	**0.050**
Patellar mode 16	0.7%	0.550 (0.321–0.943)	**0.029**	0.469 (0.280–0.786)	**0.004**
Patellar mode 17	0.7%	0.580 (0.353–0.953)	**0.032**	0.631 (0.378–1.052)	0.078
Tibial mode 15	1.3%	1.643 (1.025–2.636)	**0.039**	1.497 (0.922–2.430)	0.103
Tibial mode 19	1.0%	1.718 (1.099–2.686)	**0.017**	1.714 (1.046–2.811)	**0.033**
Tibial mode 26	0.6%	0.692 (0.444–1.079)	0.105	0.613(0.392–0.960)	**0.032**

**Note:** Shape modes significantly associated with MCID non-achievement in either unadjusted or sex- and BMI-adjusted analyses are shown. Odds ratios are reported per 1-SD increase in standardized shape mode scores. Patient-level cluster-robust standard errors were used to account for bilateral knees.

**Abbreviation:** BMI, body mass index; KSS-Function, Knee Society Function Score; MCID, minimal clinically important differences; TKA, total knee arthroplasty.

### 3.5 Anatomical interpretation of MCID-associated shape modes

**Fig. 4:**
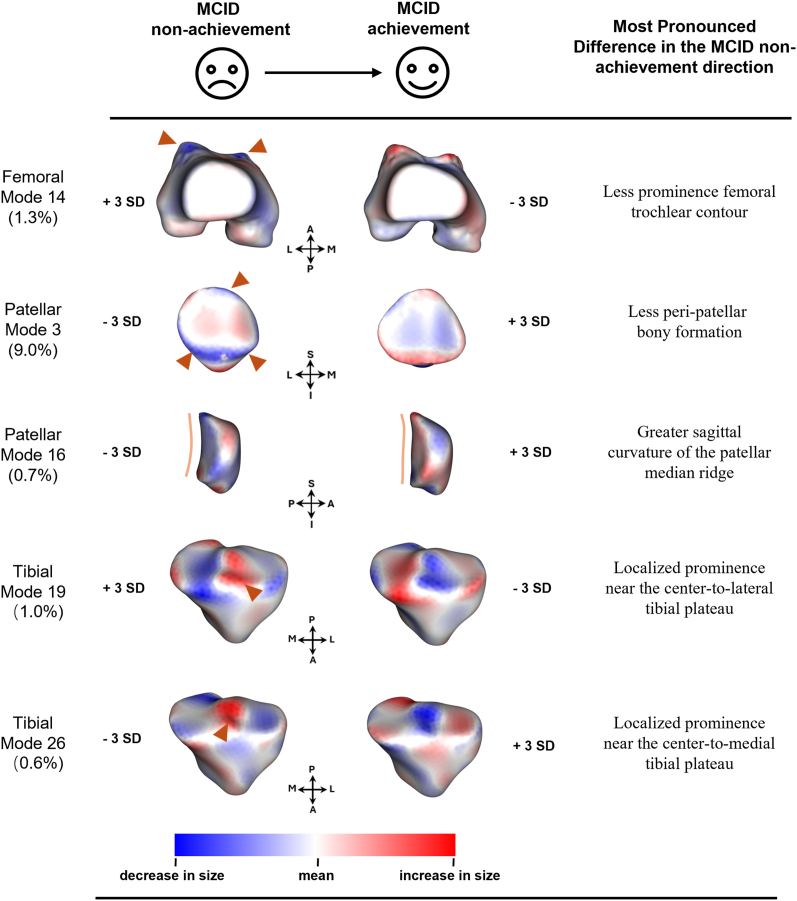
Anatomical interpretation of bone shape modes associated with 1-year KSS-Function MCID non-achievement after TKA. For each mode, the morphology corresponding to the MCID non-achievement-associated direction is shown on the left, and the opposite direction is shown on the right. Color maps indicate local surface displacement relative to the mean shape, with blue representing decreased size and red representing increased size. Dark brown arrows indicate the regions with the most pronounced anatomical differences in the MCID non-achievement-associated direction. The right column summarizes the main anatomical feature indicated by the arrows. MCID, minimal clinically important difference; SD, standard deviation; TKA, total knee arthroplasty.

As shown in Fig. 4, femoral mode 14 was associated with higher odds of MCID non-achievement and showed less bony prominence around the femoral trochlear contour along the +3 SD direction of this mode. Patellar modes 3 and 16 were associated with lower odds of MCID non-achievement. For patellar mode 3, the −3 SD morphology, corresponding to higher MCID non-achievement, showed less peri-patellar bony formation, whereas the +3 SD morphology showed a more pronounced peri-patellar bony contour. For patellar mode 16, the −3 SD morphology showed a more curved median ridge contour in the sagittal view, whereas the +3 SD morphology showed a flatter median ridge contour.

Tibial mode 19 was associated with higher odds of MCID non-achievement and showed localized surface prominence around the central-to-lateral tibial plateau near the intercondylar region in the +3 SD direction. Tibial mode 26 was associated with lower odds of MCID non-achievement; for this mode, the −3 SD morphology, corresponding to higher MCID non-achievement, showed localized surface prominence around the central-to-medial tibial plateau near the intercondylar region.

### 3.6 Incremental value of CT-derived shape features for identifying MCID non-achievement

For the internal discrimination analysis, tibial mode 19 and patellar mode 16 were retained in the parsimonious two-mode shape model, as both were significant in unadjusted and sex- and BMI-adjusted analyses with consistent effect directions. The clinical-only model included sex and BMI, the shape-only model included tibial mode 19 and patellar mode 16, and the combined model included all four predictors.

In repeated patient-level grouped cross-validated ROC analysis, the clinical-only model including sex and BMI achieved an AUC of 0.680 based on mean cross-validated predictions, with a mean repeated-CV AUC of 0.677 ± 0.019. The shape-only model, including tibial mode 19 and patellar mode 16, showed comparable discrimination, with an AUC of 0.696 and a mean repeated-CV AUC of 0.688 ± 0.016. The combined model achieved the highest discrimination, with an AUC of 0.754 and a mean repeated-CV AUC of 0.747 ± 0.021. The empirical 2.5th–97.5th percentile interval for the combined model was 0.700–0.778, compared with 0.625–0.704 for the clinical-only model and 0.644–0.712 for the shape-only model (Table 4).

**Table 4:** Cross-validated discriminative performance of clinical, shape, and combined models for 1-year KSS-Function MCID non-achievement after TKA.

Model	Included variables	AUC from mean CV prediction	Repeated-CV AUC

Clinical-only	Sex, BMI	0.680	0.677 ± 0.019 (0.625–0.704)
Shape-only	Tibial mode 19	0.696	0.688 ± 0.016 (0.644–0.712)
	Patellar mode 16		
Combined	Sex, BMI	0.754	0.747 ± 0.021 (0.700–0.778)
	Tibial mode 19		
	Patellar mode 16		

**Note:** MCID non-achievement was coded as 1 and MCID achievement as 0. Discriminative performance was evaluated using repeated five-fold patient-level stratified grouped cross-validation, with patient ID used as the grouping variable to avoid patient-level leakage from bilateral knees. AUC from mean CV prediction was calculated using the mean out-of-fold predicted probability for each knee across 100 repeated cross-validation runs. Repeated-CV AUC is presented as mean ± standard deviation across the 100 repeated cross-validation runs, with the empirical interval representing the 2.5th–97.5th percentile range of repeated-CV AUCs.

**Abbreviation:** AUC, area under the receiver operating characteristic curve; BMI, body mass index; CV, cross-validation.

In paired patient-level bootstrap analysis, the observed AUC difference between the combined and clinical-only models was 0.074. The bootstrap mean ΔAUC was 0.072, with a 95% CI of −0.022 to 0.164 and a two-sided bootstrap P value of 0.130 (Table S2).

## 4 Discussion

This study evaluated whether preoperative CT-derived 3D bone shape features were associated with 1-year KSS-Function MCID non-achievement after TKA. Our findings indicate that specific femoral, tibial, and patellar shape modes were associated with failure to achieve clinically meaningful functional improvement, even after adjustment for clinical covariates. In the internal discrimination analysis, the combined model integrating clinical data and bone shape features showed a numerically higher cross-validated AUC than the clinical-only model, although the bootstrap comparison of AUC difference did not reach statistical significance. These findings suggest that CT-derived 3D bone shape may capture an underrecognized anatomical dimension related to postoperative functional recovery after TKA.

In our analysis, female sex and higher BMI were associated with a greater likelihood of failing to achieve the MCID after TKA. These findings align with previous studies reporting that sex and obesity-related factors may influence patient-reported outcomes and functional recovery after TKA. In contrast, while prior studies have indicated that preoperative functional status affects the probability of achieving MCID [27], we found no significant association with preoperative KSS-Function scores. This discrepancy may reflect differences in outcome instruments, MCID definitions, or cohort characteristics. Furthermore, the relatively small number of MCID non-achievement events in our cohort may also have limited the statistical power to detect more modest associations.

Several bone shape patterns associated with MCID non-achievement were consistent with prior evidence linking OA severity to postoperative improvement. Femoral mode 14 and patellar mode 3 both indicated less pronounced patellofemoral bony remodeling in the MCID non-achievement-associated direction, with less bony prominence around the femoral trochlea and patella, respectively. This may reflect less advanced patellofemoral osteoarthritic change, consistent with studies showing that patients with more advanced OA may experience greater symptomatic and functional gains after arthroplasty [15,28]. However, because osteophyte burden and patellofemoral OA severity were not directly quantified in this study, this interpretation should be considered exploratory.

Beyond OA-severity-related remodeling, our analysis also identified localized three-dimensional anatomical variations involving the patella and central tibial plateau. Patellar mode 16 reflected variation in the sagittal curvature of the patellar median ridge. Because all knees underwent Mako robotic-assisted TKA without patellar resurfacing in our cohort, the more curved median ridge morphology associated with MCID non-achievement raises the possibility that native patellar shape may influence geometric compatibility with the standardized femoral implant trochlea [29]. The tibial modes provided a complementary information: modes 19 and 26 both involved contour variation near the central tibial plateau and intercondylar region, with mode 19 centered more central-to-lateral near the intercondylar eminence and mode 26 showing a more central-to-medial surface prominence resembling an osteophyte-like or “anvil”-type morphology [30]. These findings suggest that CT-derived SSM may capture localized anatomical phenotypes not readily represented by conventional radiographic grading. Further studies incorporating finite element analysis or postoperative patellar tracking assessment are needed to determine whether these morphological features are related to implant-patella contact stress and functional recovery after TKA [31].

Our study had several limitations. First, all surgeries were performed using a single robotic-assisted knee arthroplasty system under a consistent functional alignment philosophy. This minimized procedural variability but also limits the generalizability of our findings. Second, our study was based on CT imaging, which provides high spatial resolution for elucidating bony anatomy but also involves higher radiation exposure than conventional radiographs [32]. Further investigation into low-dose CT approaches, such as weight-bearing CT [33], and the potential integration of multi-view radiographs may help translate these 3D insights into broader clinical implementation. Third, although KSS-Function score is widely used [12], it primarily reflects functional recovery and does not directly measure patient satisfaction. In addition, because MCID non-achievement is based on change from baseline, this endpoint may be influenced by baseline functional status and ceiling effects. Broader patient-reported outcome measures, such as Oxford Knee Score [34] and Short Form-36 Health Survey [35], capture other aspects of postoperative outcomes including pain, mental well-being, and quality of life. Future studies incorporating broader patient-reported outcome measures and direct satisfaction assessments are needed to determine whether the identified bone shape features are also associated with more comprehensive patient-perceived outcomes. Finally, this was a single-center study with a relatively modest sample size and limited number of MCID non-achievement events, and external validation in larger and more diverse cohorts is needed.

In conclusion, three-dimensional preoperative bone shape provides meaningful insights into patient functional outcomes after TKA. The associated femoral, tibial, and patellar modes suggest that clinically meaningful functional improvement may be related to joint-level anatomical variation not fully captured by conventional clinical assessment. When added to clinical data, bone shape features provided additional predictive information, but the improvement in cross-validated discrimination was not statistically robust in this study. Therefore, while CT-derived 3D bone shape analysis may offer complementary anatomical insights for patient stratification, its additive value beyond clinical factors requires confirmation in larger, adequately powered cohorts.

## Author contributions

WW, LCML, PKC and CYW conceptualized and designed the research. TSJ, WW, HF and KYC collected data. WW, TSJ, ZQW and LCC analyzed results. LCML, TML, DHJ, JAL, HF and KYC contributed to results interpretation. WW and LCML wrote the original draft. TML, DJH, JAL, TSJ, ZQW and CYW reviewed and edited the paper. PKC and CYW supervised the project.

## Declaration of generative AI in scientific writing

During the preparation of this work, the authors used ChatGPT (OpenAI) to assist with improving language clarity and grammatical expression. After using this tool, the authors reviewed and edited the content as needed and take full responsibility for the content of the publication.

## Role of the funding source

This study is supported by the RISA seed fund (P0043002, P0051049, P0050709) and 10.13039/501100022097RIAM seed fund (P0050824), Mainland/10.13039/100027891GBA Research Funding Scheme (P0049195), Innovation & Technology Fund for Better Living (FBL/B046/22/S), and RGC CRF grant (C5117-25GF).

## Conflict of interest

DJH reports consultancy fees from Pfizer, Lilly, TLCBio, and Novartis, and serves as Editor in Chief of *Osteoarthritis and Cartilage* and Section Editor for osteoarthritis for UpToDate. He is a member of the Data Safety Monitoring Boards for the Success trial for spinal stenosis (ACTRN12617000884303) and ICM-203 for knee osteoarthritis (NCT04875754) and is a Board Member of the Osteoarthritis Research Society International. CYW serves as Associate Editor of Osteoarthritis and Cartilage and is a Board Member of the Osteoarthritis Research Society International. Given his role as Guest Editor of *Osteoarthritis and Cartilage Open*, CYW had no involvement in the peer-review of this article and has no access to information regarding its peer-review. Full responsibility for the editorial process for this article was delegated to another journal editor.
