# Supplementary material for: CT-based 3D bone shape modes are associated with clinically meaningful functional improvement after total knee arthroplasty
Source: Osteoarthr Cartil Open. 2026 Jun 6;8(3):100839. doi: 10.1016/j.ocarto.2026.100839 (PMC13276586; doi:10.1016/j.ocarto.2026.100839)
Supplement: Multimedia component 1 [file mmc1.docx]

**Supplementary material**

**List of Supplementary Figures and Tables:**

- **Figure S1:** Region of interest in the distal femur and proximal tibia
- **Table S1.** The characteristics of knees for statistical shape modeling
- **Table S2.** Patient-level bootstrap comparison of AUC between the clinical-only and combined models


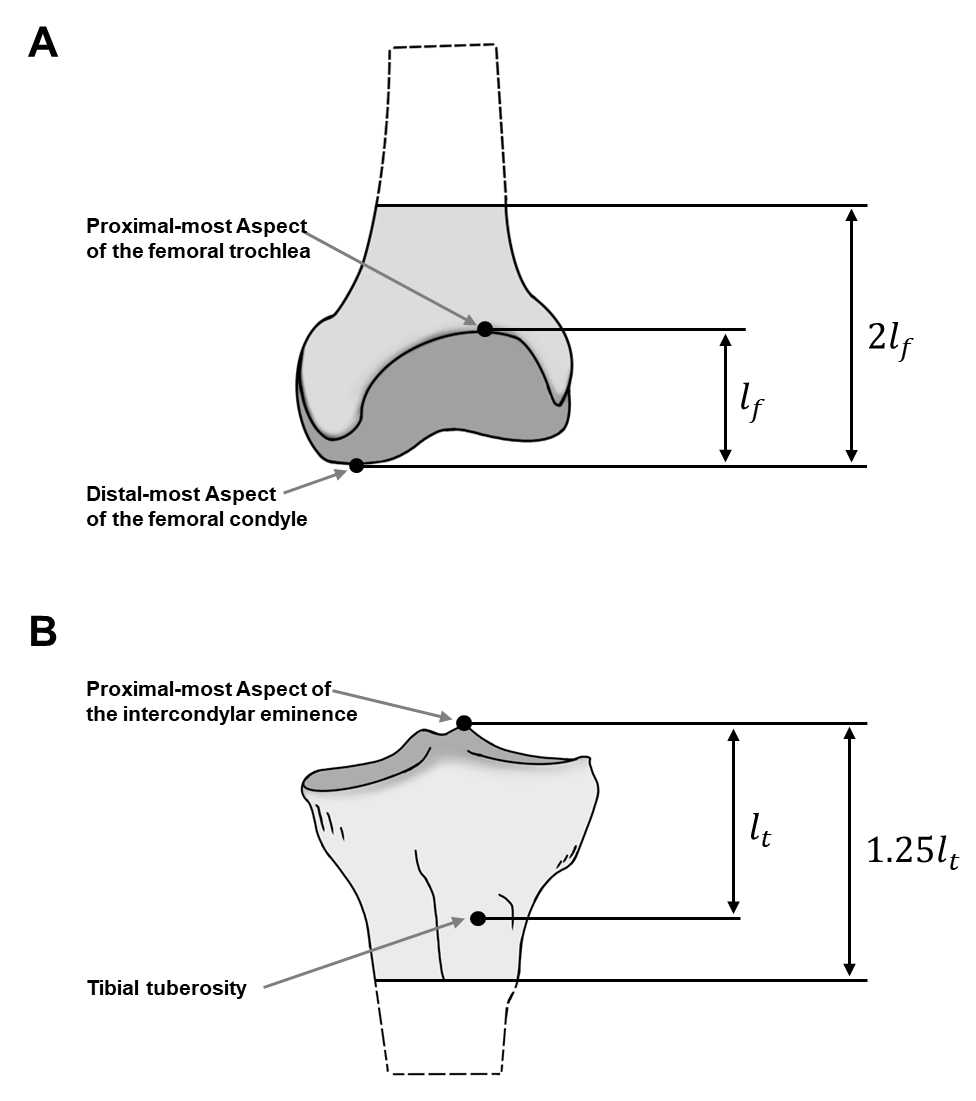


**Figure S1**: Region of interest in distal femur and proximal tibia. A, femur surfaces were transected at a 2.0 ratio, proportionate to the vertical distance from the proximal-most aspect of the femoral trochlea to the distal-most aspect of the medial femoral condyle ($l_{f}$). B, tibia surfaces were transected at a 1.25 ratio, proportionate to the vertical distance from the proximal-most aspect of the intercondylar eminence to the tibial tuberosity ($l_{t}$).

| **Table S1.** The characteristics of knees for statistical shape modeling | |
| --- | --- |
| Age at surgery, year | 67.5 ± 6.5 (51.2-85.6) |
| Knees, n (female sex) | 258(62.0%) |
| Height, cm | 157.4 ± 8.6 (120.0 - 186.0) |
| Weight, kg | 67.9 ± 12.6 (31.7 - 110.0) |
| BMI, kg/m^2^ | 27.4 ± 4.6 (16.8 - 44.6) |
| Preop KSS-Knee | 57.7 ± 16.5 (0 - 94) |
| Preop KSS-Function | 49.8 ± 12.8 (0 - 85) |
| **Note:** Data are reported as n (%) or mean ± SD, with the range in parentheses. Height and BMI are unavailable for 3 knees.  **Abbreviation:** TKA, total knee replacement; BMI, Body Mass Index, KSS-Knee, Knee Society Knee Score, KSS-Function: Knee Society Function Score. | |

| **Table S2.** Patient-level bootstrap comparison of AUC between the clinical-only and combined models | | | | |
| --- | --- | --- | --- | --- |
| Comparison | AUCs and observed ΔAUC | Bootstrap mean ΔAUC | Bootstrap 95%C | P value |
| Combined vs. clinical-only | 0.754 vs 0.680 (0.074) | 0.072 | [-0.022, 0.164] | 0.130 |
| **Note:** AUCs were calculated using mean cross-validated predicted probabilities. ΔAUC was defined as the AUC of the combined model minus the AUC of the clinical-only model. Bootstrap confidence intervals and P values were calculated using paired patient-level bootstrap resampling with 5,000 iterations, with patients used as the resampling unit to preserve the correlation structure of bilateral knees.  **Abbreviation:** AUC, area under the receiver operating characteristic curve; CI, confidence intervals. | | | | |
